# Low-Power Two-Color Stimulated Emission Depletion Microscopy for Live Cell Imaging

**DOI:** 10.3390/bios11090330

**Published:** 2021-09-10

**Authors:** Jia Zhang, Xinwei Gao, Luwei Wang, Yong Guo, Yinru Zhu, Zhigang Yang, Wei Yan, Junle Qu

**Affiliations:** Key Laboratory of Optoelectronic Devices and System of Ministry of Education and Guangdong Province, College of Physics and Optoelectronic Engineering, Shenzhen University, Shenzhen 518060, China; julyzhang2021@163.com (J.Z.); 1910454012@email.szu.edu.cn (X.G.); wanglowell@szu.edu.cn (L.W.); 1800284004@email.szu.edu.cn (Y.G.); 1900453009@email.szu.edu.cn (Y.Z.); zhgyang@szu.edu.cn (Z.Y.)

**Keywords:** stimulated emission depletion, low power STED, super-resolution microscopy, dynamic live cell imaging

## Abstract

Stimulated emission depletion (STED) microscopy is a typical laser-scanning super-resolution imaging technology, the emergence of which has opened a new research window for studying the dynamic processes of live biological samples on a nanometer scale. According to the characteristics of STED, a high depletion power is required to obtain a high resolution. However, a high laser power can induce severe phototoxicity and photobleaching, which limits the applications for live cell imaging, especially in two-color STED super-resolution imaging. Therefore, we developed a low-power two-color STED super-resolution microscope with a single supercontinuum white-light laser. Using this system, we achieved low-power two-color super-resolution imaging based on digital enhancement technology. Lateral resolutions of 109 and 78 nm were obtained for mitochondria and microtubules in live cells, respectively, with 0.8 mW depletion power. These results highlight the great potential of the novel digitally enhanced two-color STED microscopy for long-term dynamic imaging of live cells.

## 1. Introduction

STED is the earliest super-resolution imaging approach to break the optical diffraction limit among many super-resolution imaging technologies [1,2,3,4,5,6]. It has attracted considerable interest owing to its merits including high imaging speed, high spatial resolution, and large imaging depth. STED shrinks the point spread function (PSF) of excited light via the stimulated emission effect, resulting in the breakage of the optical diffraction limit and nanometer-scale spatial resolution [7]. Usually, to effectively force the fluorophore into a dark state and achieve the compression of the PSF, the excitation light must reach the sample approximately 200 ps before the depleted light (STED light), and the stimulated emission process has to precede the spontaneous emission. Spontaneous emission typically occurs within a few nanoseconds (on the order of fluorescence lifetime of the fluorophore) after the sample is excited. Therefore, the stimulated emission process has to be completed within this time period. A very high STED light power is required to completely deplete the fluorescence in the overlapping areas due to the short time range and the small stimulated emission cross-section of the fluorescent molecule. In addition, high-resolution results require extremely high STED light power because the spatial resolution of STED imaging is proportional to the power of STED light [8]. Therefore, photobleaching [9] and phototoxicity [10] often occur when high spatial resolution is made possible. This severely limits the application and development of STED for live cell imaging.

Numerous efforts have been made to address this issue. For instance, in 2013, S. W. Hell’s group proposed the use of time-gated detection technology to improve resolution at low energy [10]. Adaptive optics aberration correction technology was also offered as an effective method [11,12,13]. In 2013, Kuang et al. proposed a super-resolution imaging method similar to STED, namely fluorescence emission difference (FED) microscopy, which could achieve low light power super-resolution imaging [14]. Our research group also developed a variety of methods to improve the imaging resolution and quality at low energy. For example, a phasor plot approach was applied to separate photons from fluorescence lifetime imaging (FLIM) and STED images and realized low power STED super-resolution imaging [15]. A novel deconvolution method virtual single-pixel imaging (v-SPI) was developed in 2020 [16]. In particular, the digitally enhanced STED (DE-STED) technology was proposed to achieve a resolution of one-eighth wavelength under ultralow depletion optical power [17]. All the proposed methods relaxed the restriction on fluorophores due to phototoxicity and photobleaching of high depletion power.

Although STED super-resolution imaging has been applied in the field of biomedical photonics, two-color live cell STED super-resolution imaging still cannot be widely used due to the complexity of the system, high cost, and high laser power. Early two-color STED microscope systems were very complicated, attributed to STED microscopes requiring four precisely aligned laser beams (two for fluorescence excitation and two for fluorescence quenching by stimulated emission), which poses special challenges for imaging multiple fluorophores [18,19]. In order to reduce the cost and complexity of the two-color system, the two fluorophores with partial overlapping emission spectra and a long Stokes redshift between the excitation and emission spectra are often used as two-color STED probes [20]. In this case, two fluorophores are depleted by a single STED beam, and two different excitation beams are used to achieve color separation. At present, the same concept has been extended to three [21,22] and four [23] fluorophores. In 2013, S. W. Hell’s group proposed a new two-color STED system with three pulsed lasers (two excitation lasers and one depletion laser) for Atto594 and KK14 fluorescent dyes [24]. In addition, Tønnesen et al. developed a two-color STED method using only two pulsed lasers (one excitation laser and one depletion laser), which used green-yellow fluorescent dyes [25]. Furthermore, two-color STED microscopy has been achieved by measuring the fluorescence lifetime [26] or using light-switched fluorescent proteins [27]. In 2020, our group proposed a new method to achieve two-color STED imaging with only one laser source (supercontinuum pulsed laser) [28]. Although these multicolor STED methods can reduce cost and system complexity, they still face the problem of excessively high laser power.

Therefore, in this study, we combined the digital enhancement method [17] with our home-built one-laser two-color STED system to realize low-power two-color DE-STED microscopy for dynamic live cell imaging. In the DE-STED method, a donut image is first obtained by subtracting the STED image from the confocal image. Then, the donut image is digitally enhanced to further obtain higher resolution. The DE-STED image is obtained by subtracting the enhanced donut image from the original confocal image multiplied by an enhancement coefficient. In two-color DE-STED microscopy, we can multiply the enhancement factor in both channels to obtain the two-color DE-STED image to realize two-color super-resolution imaging at ultralow power.

## 2. Materials and Methods

### 2.1. Two-Color STED System

**Figure 1:**
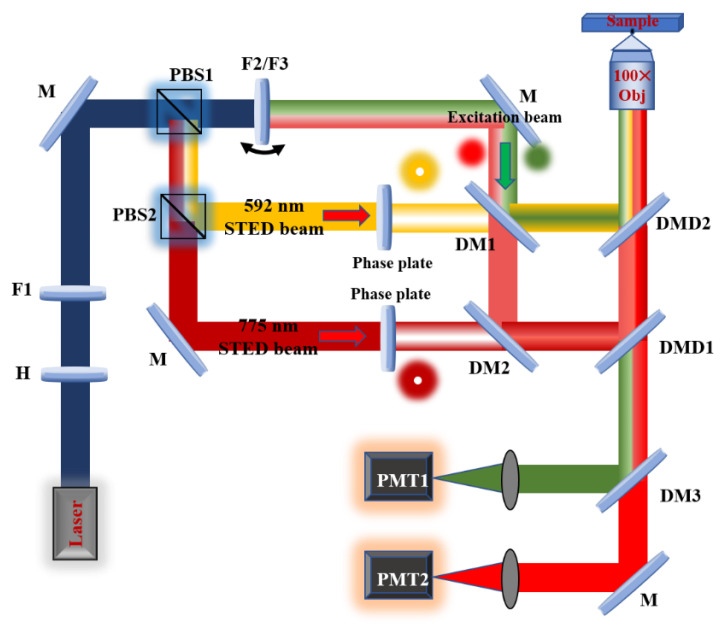
Schematic of two-color STED system.

The system diagram is shown in Figure 1. The light source is a supercontinuum pulsed laser (SC-Pro, YSL Inc., Wuhan, China) whose output wavelength is continuously adjustable from 400 to 2400 nm, and the maximum output power is 8 W. The laser is collimated and output through a single-mode fiber and then modulated into linearly polarized light using a polarizer (H). Next, the output light wavelength is adjusted within the visible light range using the filter F1. Afterward, the optical path is divided into the excitation optical path and the depletion optical path through the first polarization beam splitter (PBS1). To reduce the cost and complexity of the system, a rotatable circular wheel bracket is used to install two excitation light filters (F2/F3). Therefore, the same delay line can be served for the two channels of excitation light paths. It is easier to modulate the light path because one channel of the excitation light will be collimated along with the other channel.

An important requirement of high-resolution STED is the quality of the donut-shaped PSF, especially the zero-strength area in the center. In our two-color optical path, we customize the vortex plate (VPP) and the quarter-wave plate (QWP) of the corresponding wavelength for the specific depletion light wavelength (592 and 775 nm). The customized QWPs (QBT10-592B and QWP10-775B, LBTEK Inc., Changsha, China) ensure that each depleted beam becomes a circularly polarized beam, which is necessary to produce a donut-shaped beam combined with VPP. The most difficult and important part of the system construction is the overlap between the two excitation lasers and the two depletion lasers. Here, four dichroic mirrors (DM1, DM2, DMD1, and DMD2) are ingeniously selected, the information of dichroic mirrors is shown in Table 1. Their functions are to overlap the four lasers perfectly in the two-color channels before entering the galvanometer; after that, the excitation and depletion beams are focused on the sample by the objective lens. Another function of dichroic mirrors is to ensure that the excited two-color fluorescence signal can reach the detector port on the left in the form of transmission. The function of DM3 is to separate the emission fluorescence signals excited by the 488 and 635 nm lasers. Due to the particularity of its transmission spectrum, all fluorescent signals below 550 nm can be reflected to the photomultiplier tube PMT1, and fluorescent signals with a wavelength longer than 550 nm reach PMT2.

### 2.2. Method of Two-Color DE-STED

**Figure 2:**
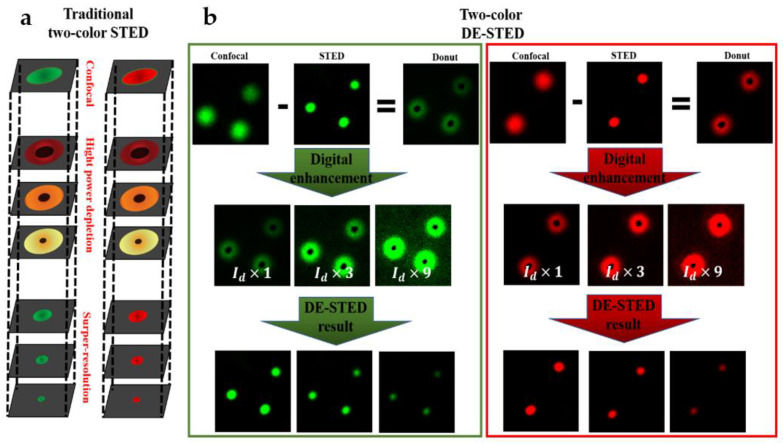
Schematic principles of two-color DE-STED. (**a**) The principle of traditional two-color STED. (**b**) The principle of two-color DE-STED.

According to STED theory, the resolution has no upper limit [10]. However, the actual limit lies in the available laser power, dyes, and optical precision to produce a donut-shaped depleted beam with zero central light intensity [15]. In a traditional two-color STED system, two beams of excitation light (488 and 635 nm) are used to make the ground state particles jump to the excited state, and the other two beams of donut-shaped beams (STED light: 592 and 775 nm) are used to irradiate the sample and cause the stimulated radiation effect so that the fluorescent molecules on the focal spot are depleted by the STED light and lose the ability to emit fluorescence. The light-emitting area that can emit fluorescence is compressed by limiting the stimulated radiation area and obtaining a light-emitting point smaller than the diffraction limit. As shown in Figure 2a, a smaller PSF is achieved by depleting the photons with the donut-shaped STED beam in traditional STED imaging, and we can see that the zero-intensity area of the STED beam decreases with increasing depletion power and generates a smaller fluorescent spot. The lateral resolution of the STED is determined by the formula $\Delta x=\lambda /2NA\sqrt{1+I_{STED}/I_{sat}}$, where λ is the wavelength of the excitation light, NA is the numerical aperture of the objective, $I_{STED}$ is the intensity of the STED laser, and $I_{sat}$ is the saturation intensity of stimulated radiation. According to this equation, the resolution can be improved by increasing the intensity of the STED beam or reducing the saturation stimulated radiation intensity of the probe directly. However, $I_{sat}$ depends only on the characteristics of the material, including the stimulated emission cross-section and fluorescence lifetime of the fluorescent probe [29]. According to the resolution function of STED, a high resolution can be obtained when the depletion beam is strong enough.

In the two-color DE-STED, the STED image is subtracted from the confocal image to generate a donut image composed of depletion photons, and the confocal and STED images of two colors are obtained from the same imaging position.

(1) $${\mathrm{PSF}}_{\mathrm{Donut}\;\mathrm{Green}}={\mathrm{PSF}}_{\mathrm{Confocal}\;\mathrm{Green}}-{\mathrm{PSF}}_{\mathrm{STED}\;\mathrm{Green}}$$

(2) $${\mathrm{PSF}}_{\mathrm{Donut}\;\mathrm{Red}}={\mathrm{PSF}}_{\mathrm{Confocal}\;\mathrm{Red}}-{\mathrm{PSF}}_{\mathrm{STED}\;\mathrm{Red}}$$

where ${\mathrm{PSF}}_{\mathrm{Confocal}},{\;\mathrm{PSF}}_{\mathrm{STED}}$, and ${\mathrm{PSF}}_{\mathrm{Donut}}$ are the PSF of confocal, STED, and Donut; Green and Red stand for channels of 488 and 635 nm.

The digital enhancement method can be used to realize the process of increasing the depletion power in the traditional STED to improve depletion efficiency. Two-color donut images (592 and 775 nm) are obtained by subtraction (the confocal image minus the STED image) and then multiplied by the *k* factor (greater than 1), which is akin to enhancing the power of the depletion light. In this way, a donut image with a small central area can be obtained. After subtracting the enhanced (multiplied by a *k* factor) donut image from the original confocal image, a DE-STED image with higher resolution can be obtained, as shown in Figure 2b.

(3) $${\mathrm{PSF}}_{\mathrm{DE}-\mathrm{STED}\;\mathrm{Green}}={\mathrm{PSF}}_{\mathrm{Confocal}\;\mathrm{Green}}-k_{1}\times {\mathrm{PSF}}_{\mathrm{Donut}\;\mathrm{Green}}$$

(4) $${\mathrm{PSF}}_{\mathrm{DE}-\mathrm{STED}\;\mathrm{Red}}={\mathrm{PSF}}_{\mathrm{Confocal}\;\mathrm{Red}}-k_{2}\times {\mathrm{PSF}}_{\mathrm{Donut}\;\mathrm{Red}}$$

where ${\mathrm{PSF}}_{\mathrm{DE}-\mathrm{STED}}\;\mathrm{is}$ the PSF of DE-STED, *k* is the enhancement factor.

In this method, the center zero-intensity area of the donut-shaped beam decreases with increasing *k*, and a higher resolution image is obtained. This method is flexible and has a better two-color super-resolution result, even if the *k* factors of the two-color channels are different if the value of *k* is larger than 1.

## 3. Results

### 3.1. Fluorescent Bead Imaging

**Figure 3:**
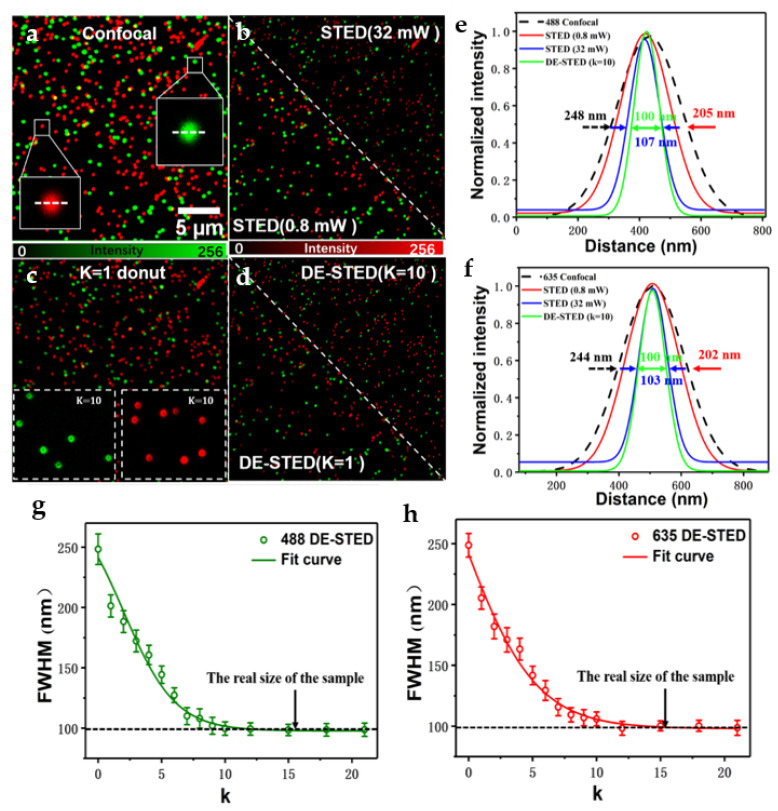
Imaging results of two-color DE-STED for fluorescent beads. (**a**) Two-color confocal image (green: 488 nm, red: 635 nm). (**b**) STED image with depletion power 0.8 and 32 mW. (**c**) Donut image, where the insets are donut images of *k* = 10. (**d**) DE-STED results at *k* = 1 and 10. (**e**,**f**) The resolution curves of the green and red channels for confocal, STED (0.8 and 32 mW), and *k* = 10 DE-STED, taking the marked fluorescent beads in (**a**) as the research object. (**g**,**h**) The curves of the relationship between FWHM and *k* value for the green and red channels. Scale bar: 5 μm.

The spatial resolution of the two-color DE-STED microscope was assessed by measuring the effective PSF with two kinds of 100 nm fluorescent beads (488/560 and 633/660, Thermo Fisher). Figure 3a,b,d show the confocal, STED, and DE-STED images, respectively. Figure 3e shows that when the 488 nm excitation light was 0.06 mW and the 592 nm depleted light was 0.8 mW, the maximum full widths at half maximum (FWHMs) were 248 and 205 nm for confocal and STED, respectively. The resolution improvement was not obvious because of the low depletion power. The FWHM increased to 107 nm when the depletion power was increased to 32 mW, which was close to the actual size of the beads. Therefore, to gain perfect resolution, the depletion power needed to be extremely strong in traditional STED. To acquire the DE-STED image, we used the confocal result in Figure 3a to subtract the STED image in Figure 3b and then achieved the donut image in Figure 3c. The DE-STED result in Figure 3d was obtained by subtracting the donut result in Figure 3c from the confocal result in Figure 3a with *k* = 1 and *k* = 10. The resolution of the fluorescent beads was significantly improved with increasing *k* value. The resolution was 102 ± 7 nm when *k* = 10, which was obtained from the average FWHM of 10 typical fluorescent beads (approximate the resolution of 32 mW STED power), and close to the actual size of the beads itself (see Figure 3g). Due to the limitation of the actual size of the beads, the resolution stopped improving with increasing *k* value when *k* ≥ 10. The same result was observed in the 635 nm channel. When the 635 nm excitation light was 0.06 mW and the 775 nm depletion light was 0.8 mW, the FWHMs of confocal and STED were 244 and 202 nm, respectively. The resolution was increased to 103 nm when the depletion power was higher than 32 mW, as shown in Figure 3f. When processed by the DE-STED method, the average FWHM of 10 fluorescent beads was 106 ± 5 nm at *k* = 10, which approached the resolution of 32 mW depletion power in Figure 3b. As shown in Figure 3h, the resolution does not continue to improve when *k* ≥ 10 because the FWHM is close to the actual size of the fluorescent beads. The above results showed that the DE-STED method with *k* = 10 at 0.8 mW depletion power can obtain a considerable resolution of STED at 32 mW depletion power. It follows from this that the two-color DE-STED is a powerful imaging method for low-power high-resolution imaging.

### 3.2. Fixed Cell Imaging

**Figure 4:**
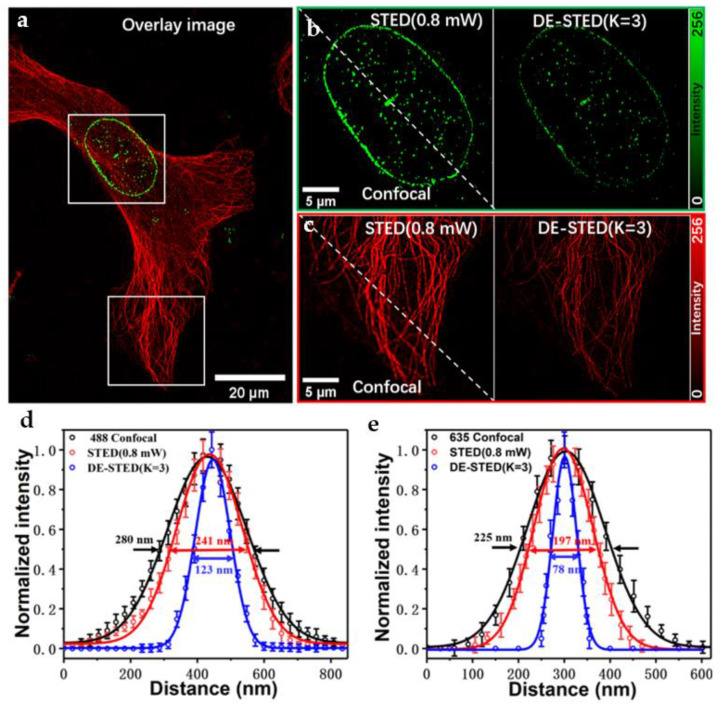
Two-color DE-STED imaging results from the same fixed U2OS cell. (**a**) Merged confocal images of two-color channels including nuclear membrane (green) and microtubules (red). The white frame area in the figure is the research object. (**b**) Confocal, STED (0.8 mW), and DE-STED (*k* = 3) images of the nuclear membrane in the green channel. (**c**) Confocal, STED (0.8 mW), and DE-STED (*k* = 3) images of the microtubule in the red channel. (**d**) The average FWHM of the nuclear membrane in the confocal, STED (0.8 mW), and DE-STED (*k* = 3) images in (**b**). (**e**) The average FWHM of microtubule in the confocal, STED (0.8 mW), and DE-STED (*k* = 3) images in (**c**). Scale bars: 20 and 5 μm.

In order to show the power of the two-color DE-STED, we performed STED imaging of human osteosarcoma cells (U2OS). Cells were labeled with immunostaining, and the nuclear membrane and microtubule were labeled with Alexa Fluor 488 (150077, Abcam, UK) and Atto 647N (50185, Sigma-Aldrich, Burlington, MA, USA), respectively. Two-color DE-STED results of fixed U2OS cells are shown in Figure 3, which includes green channel and red channel. Figure 4a shows the two-color confocal image of the nuclear membrane and microtubule tissue of the cell in a larger field of view. Figure 4b,c show the STED and DE-STED images of the enlarged areas in Figure 4a, respectively. First, two channels were researched separately, as shown in Figures S1–S4. For the green channel (excitation wavelength 488 nm at power of 84 μW), Figure S1a–d shows images under confocal and three different depletion powers (0.8, 16, and 32 mW), and Figure S1e,f shows normalized intensity curves of the white underlined part. The FWHM of the confocal was 278 nm. When the depletion power was 0.8, 16, and 32 mW, the resolution of STED was 241, 183, and 123 nm, respectively. Regrettably, although better resolution can be obtained under high depletion power, the intensity of the imaging dropped sharply and induced irreversible bleaching damage to the biological samples. To address this problem, we performed a DE-STED experiment, and the results are shown in Figure S2. Comparing Figure S2b and Figure S2a, there is no obvious difference under low depletion power. The donut image of Figure S2c can be obtained by subtracting Figure S2b from Figure S2a when the value of *k* is 1. Figure S2d shows the DE-STED images at different *k* values. The intensity of the donut image becomes significantly stronger when the *k* value is equal to 3, and it achieved a resolution of 123 nm, as shown in Figure S2e, which is equivalent to the effect with a depletion power of 32 mW in Figure S1. As shown in Figure S2d,e, the FWHM is gradually improved with increasing *k* value. However, the resolution was not improved further when the value of *k* was greater than 3 and was eventually restricted to approximately 120 nm. Although the resolution of DE-STED images can be improved by increasing the value of *k*, the signal intensity may decrease rapidly with increasing enhancement coefficient *k*, leading to the deterioration of the quality of super-resolution images (as shown in the white amplification area in Figure S2d). For the red channel (excitation wavelength 635 nm at a power of 68 μW), similar experimental patterns can be observed (see Figures S3 and S4). Similar to the green channel, when the value of *k* increased to a certain extent (*k* > 3), the resolution did not continue to improve, but the signal intensity decreased rapidly, resulting in poor image quality, which is no longer suitable for DE-STED. Therefore, through the above results, we observed that the two channels can both respond to the DE-STED method at low power (0.8 mW), and the obtained results can completely replace the results of the high power (32 mW) STED.

Figure 4d,e represents the FWHM of the different organelles of the two channels from the average of 10 different places. When the 488 nm excitation power was 84 μW and the 592 nm depletion power was 0.8 mW, the FWHM of the white box area of nuclear membranes in the confocal image was 280 nm, and the STED resolution was 241 nm. Although there was no obvious super-resolution effect where the depletion power was 0.8 mW compared with confocal, the resolution of DE-STED can reach 123 nm with *k* = 3, which is better than the spatial resolution at 32 mW depletion power (Figure S1). For the red channel, when the excitation power was 68 μW and the depletion power was 0.8 mW, the FWHM of the microtube of confocal mode was 225 nm. The STED resolution was 197 nm since the depletion power was only 0.8 mW. The resolution of DE-STED reached 78 nm when *k* = 3. Therefore, compared with the traditional two-color STED, the low-power two-color DE-STED showed a considerable advantage in improving spatial resolution at low depletion power by the *k* factor.

The resolution of DE-STED images can be improved greatly with increasing *k* value under a lower depletion power. However, this will not further improve the spatial resolution, which eventually will be limited to approximately one-eighth of the wavelength when the value of *k* is greater than 3 (Figures S2 and S4). Although using higher depletion power can improve the resolution of DE-STED images with smaller *k* values, the signal intensity will rapidly decrease as the depletion power increases. Therefore, low depletion power and a large *k* value are the best choices for obtaining high SNR super-resolution images. When the optimal resolution is obtained in DE-STED with 0.8 mW depletion power, the optimal *k* value is 3.

### 3.3. Live Cell Dynamic Imaging

**Figure 5:**
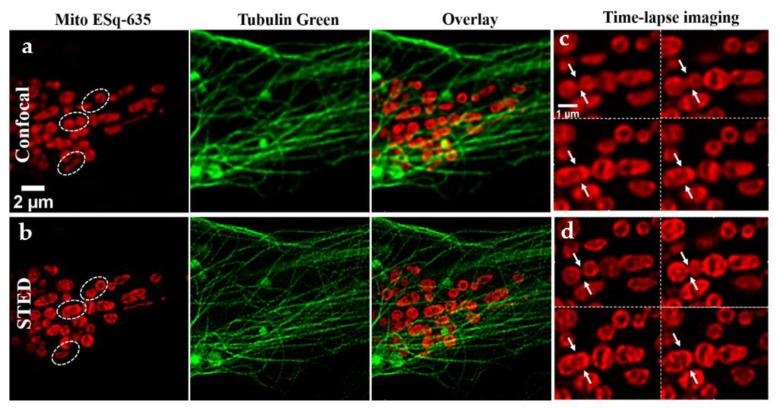
Long-term dynamic two-color imaging of U2OS cell. (**a**) Confocal image of mitochondria, tubulin, and their overlay. (**b**) STED image of mitochondria, tubulin, and their overlay after 12 min. (**c**) Confocal image of a typical mitochondrial fusion process, as indicated by the white arrow. (**d**) STED image of a typical mitochondrial fusion process, 3 min per frame. Scale bars: 2 and 1 μm.

The advantage of DE-STED is that it can achieve super-resolution imaging with low depletion power, which is particularly important in live cell imaging applications. Tubulin and mitochondria were labeled with a commercial probe named Tubulin Green (KTC4100, Abbkine) and aromatic acid dye (MitoESq-635, a red mitochondrial probe developed by our research group) for live U2OS cells, respectively. It has been reported that the aromatic acid dye MitoESq-635 has the properties of low saturation intensity, high light stability, and antibleaching, making it a highly advantageous choice for long-term, high-resolution STED imaging [30]. Figure 5 shows the results of long-term two-color imaging of live cells, including the mitochondrial imaging in the 635 nm channel and microtubule imaging in the 488 nm channel. Figure 5a shows the confocal image at the beginning, and Figure 4b shows the STED image at low depletion power (0.8 mW) after 12 min. Comparing the images in Figure 5a,b, although the resolution improvement is not distinct, the extremely low power was good for relieving photobleaching and phototoxicity, which can provide long-term live cell imaging. Since the changes in microtubule organization were not particularly obvious, we mainly observed changes in mitochondrial organization, as shown in Figure S5 and the white circle area of Figure 5. In these regions, several significant morphological changes were observed in mitochondria within 12 min. One of these regions was a typical mitochondrial fusion process, and the results are shown in Figure 5c,d, which contain the confocal and STED dynamic results of mitochondria. The image was taken every three minutes. The mitochondria merged from two bubbles into one bubble, from which the fusion process can be clearly seen (white arrow). The two mitochondrial bubbles were far apart, and there was only a very thin bridge in the middle. In the second and third pictures, the bridge connecting the two bubbles gradually becomes broader until the two bubbles gradually become one (fourth picture). The whole process is completed in approximately 9–12 min (see Figure S5). It can be seen from the results that even after several cycles of coirradiation with depletion light and excitation light, the fluorescence intensity of mitochondrial imaging results did not decrease too much, which indicates that our low-power depletion light STED system inflicts weak photobleaching in the fluorescent samples.

**Figure 6:**
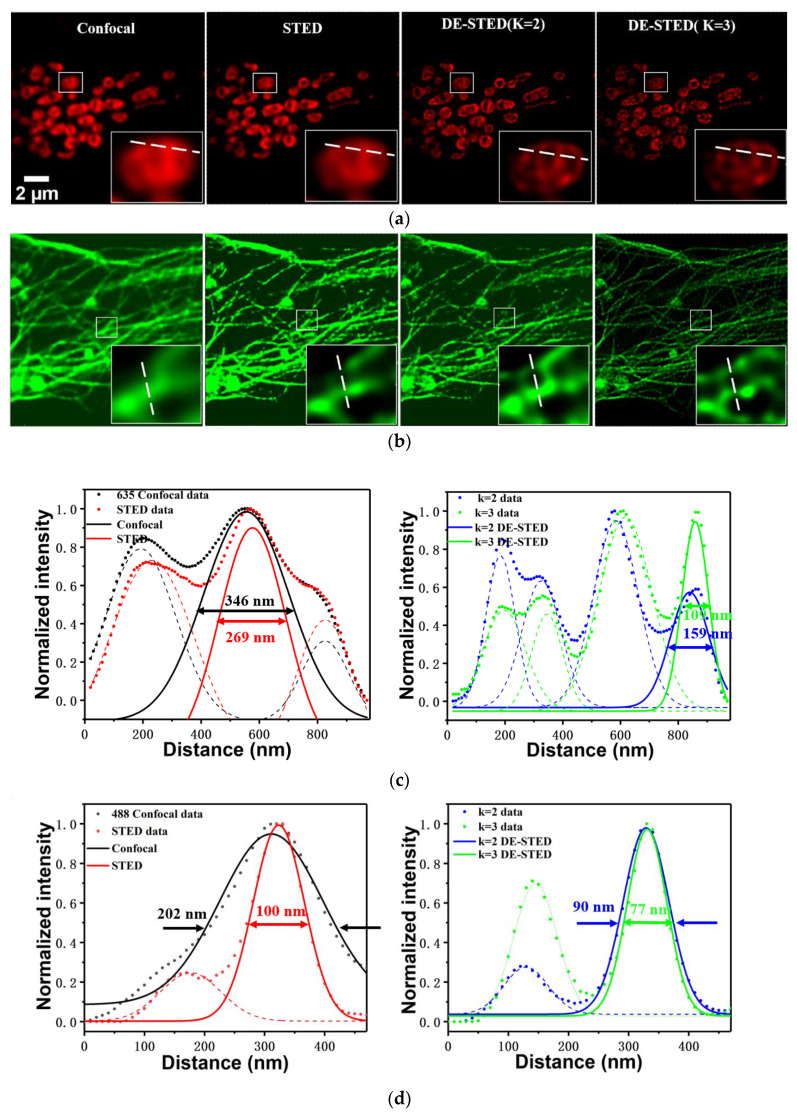
Two-color DE-STED results from the same living U2OS cell. (**a**) Confocal and STED (0.8 mW) images of mitochondria, and DE-STED images with *k* values of 2 and 3. (**b**) Confocal and STED (0.8 mW) images of tubulin, and DE-STED images with the values of 2 and 3. (**c**) Normalized intensity profiles along the white dashed lines in (**a**) for confocal (black curve), STED (red curve) image, and *k* = 2 (blue curve) and *k* = 3 (green curve) DE-STED image. (**d**) Normalized intensity profiles along the white dashed lines in (**b**) for confocal (black curve), STED (red curve) image, and *k* = 2 (blue curve) and *k* = 3 (green curve) DE-STED image. Scale bar: 2 μm.

Figure 6 shows the resolution analysis results of the two-color DE-STED live cell imaging. Figure 6a shows the confocal and STED results, and the *k* values were 2 and 3 for mitochondria. The small white box in the picture is the magnified result of a certain region of interest. Figure 6b shows the confocal and STED results, and the values of *k* values were 2 and 3 for tubulin. Figure 6c,d show the intensity curves of the white lines drawn in the white magnified boxes in Figure 6a,b, respectively, including confocal (black curve), STED (red curve), DE-STED with *k* = 2 (blue curve), and DE-STED with *k* = 3 (green curve). In the case of low-power STED in the 635 nm channel, the zoomed-in details showed that there was not much difference between confocal and STED, and the resolution improved mildly. However, there was an obvious resolution improvement for DE-STED mode, with resolutions of 159 and 109 nm for *k* = 2 and 3, respectively. The resolution was improved greatly compared with confocal and DE-STED. The same result was also obtained in the 488 nm channel. The resolutions were 202, 100, 90, and 77 nm for the confocal, STED, *k* = 2 and *k* = 3 (Figure 5d, respectively. The resolution of 77 nm is sufficient for imaging biological samples, especially with ultralow power depletion light. The same results were also observed in the nondynamic imaging of live cell samples (see Figure S6). All of the above results demonstrated the feasibility of the two-color DE-STED method and the prominent advantages for live cell imaging.

## 4. Discussion

The DE-STED method, which can realize super-resolution imaging at ultralow power, was our previous work. It achieved a spatial resolution of *λ*/8 at depletion power of 1.4 mW, which is 10 times lower than the traditional STED method [17]. However, the two-color imaging and live cell imaging experiments of the DE-STED had not been conducted. Many two-color STED systems have been invented, but in these designs, super-resolution imaging of fixed cells was only realized at high depletion power [14,19,28]. Chemical fixation often prevents the observation of molecular dynamics processes in real time [31].

Therefore, we combined the advantages of low-cost two-color STED microscopy and digital enhancement algorithms for developing low-power two-color STED microscopy. Experimental results with multiple samples showed that spatial resolution of 80 nm can be obtained with the two-color DE-STED microscope at a depletion power of 0.8 mW, which can reduce photodamage to biological samples and photobleaching of probes. Yang et al. reported that low laser power allows the cells to stay in their native state for a long time during STED imaging; the changes in mitochondrial width and fluorescence intensity under different levels of STED power were studied, and the width and fluorescence intensity changed faster when the STED power was higher [30]. To quantitate this phenomenon, George et al. measured the fluorescence intensity and photobleaching rates as a function of the laser power, and the results showed that intensity decreased exponentially under high laser irradiation, with less bleaching of the dye occurring at lower power [32]. Although quantum dots (QDs) are used as probes for STED nanoscopy imaging because of their high photoluminescence quantum yield and excellent photobleaching resistance characteristics, the downside is that they are difficult to use for live cell imaging [33,34]. We developed a two-color DE-STED system that can be used to image live cells for long-term dynamic monitoring. The fusion process of mitochondria was observed during two-color STED imaging of subcellular organelles with less than 1 mW depletion power. We obtained this result under extremely low depletion power; it was 1/10 of the optimized power of MitoEsq-635 [30] and extremely lower than MitoPB Yellow [35]. Till Stephan et al. developed live cell STED nanoscopy of mitochondrial cristae, but the imaging sequences were typically limited to 10–20 frames due to photobleaching [36]. In actual operation, different resolutions can be obtained flexibly and conveniently by performing multiplication calculations of different *k* factors. The advantages of the two-color DE-STED system include good flexibility and low cost, and it has great potential in long-term super-resolution live cell imaging. So far, two-color or multicolor long-term STED imaging of live cells is still a challenge, and the low-power two-color DE-STED microscopy is expected to provide a convenient means to solve this problem.

## Figures and Tables

**Table 1:** Information of dichroic mirrors.

	Catalog Number	Make
DM1	ZT561rdc	Chroma
DM2	ZT640rdc	Chroma
DM3	FF580	Semrock
DMD1	ZT488/647/780	Chroma
DMD2	FF395/495/610	Semrock

## Data Availability

The data presented in this study are available on request from the corresponding author.

## Supplementary Materials

The following are available online at https://www.mdpi.com/article/10.3390/bios11090330/s1, Figure S1: The comparison in resolution between confocal and STED images at different depletion powers of 488 nm channel, Figure S2: The images of nuclear envelope in a fixed U2OS cell in 488 nm channel, Figure S3: The comparison in resolution between confocal and STED images at different depletion powers in 635 nm channel, Figure S4: The imaging of tubulin in a fixed U2OS cell in 635 nm channel, Figure S5: Long-term dynamic imaging of U2OS cell mitochondria in STED mode, Figure S6: The comparison in resolution between confocal, STED, and DE-STED images of living U2OS cell.
