# Supplementary material for: Low-Power Two-Color Stimulated Emission Depletion Microscopy for Live Cell Imaging
Source: Biosensors (Basel). 2021 Sep 10;11(9):330. doi: 10.3390/bios11090330 (PMC8468006; doi:10.3390/bios11090330)
Supplement: Supplementary file 1 [file biosensors-11-00330-s001.zip › biosensors-1347927-supplementary.pdf]

biosensors

MDPI

Supporting information

Low-Power Two-Color Stimulated Emission Depletion Microscopy for Live Cell Imaging

Jia Zhang, Xinwei Gao, Luwei Wang, Yong Guo, Yinru Zhu, Zhigang Yang, Wei Yan * and Junle Qu *

Key Laboratory of Optoelectronic Devices and System of Ministry of Education and Guangdong Province, College of Physics and Optoelectronic Engineering, Shenzhen University, Shenzhen 518060, China; julyzhang2021@163.com (J.Z.); 1910454012@email.szu.edu.cn (X.G.); wanglowell@szu.edu.cn (L.W.); 1800284004@email.szu.edu.cn (Y.G.); 1900453009@email.szu.edu.cn (Y.Z.); zhgyang@szu.edu.cn (Z.Y.)
* Correspondence: weiyan@szu.edu.cn (W.Y.); jlqu@szu.edu.cn (J.Q.)

Supporting information content:

Figure S1: The comparison in resolution between confocal and STED images at different depletion powers of 488 nm channel

Figure S2: The images of nuclear envelope in a fixed U2OS cell in 488 nm channel

Figure S3: The comparison in resolution between confocal and STED images at different depletion powers in 635 nm channel

Figure S4: The imaging of tubulin in a fixed U2OS cell in 635 nm channel

Figure S5: Long-term dynamic imaging of U2OS cell mitochondria in STED mode

Figure S6: The comparison in resolution between confocal, STED, and DE-STED images of living U2OS cell

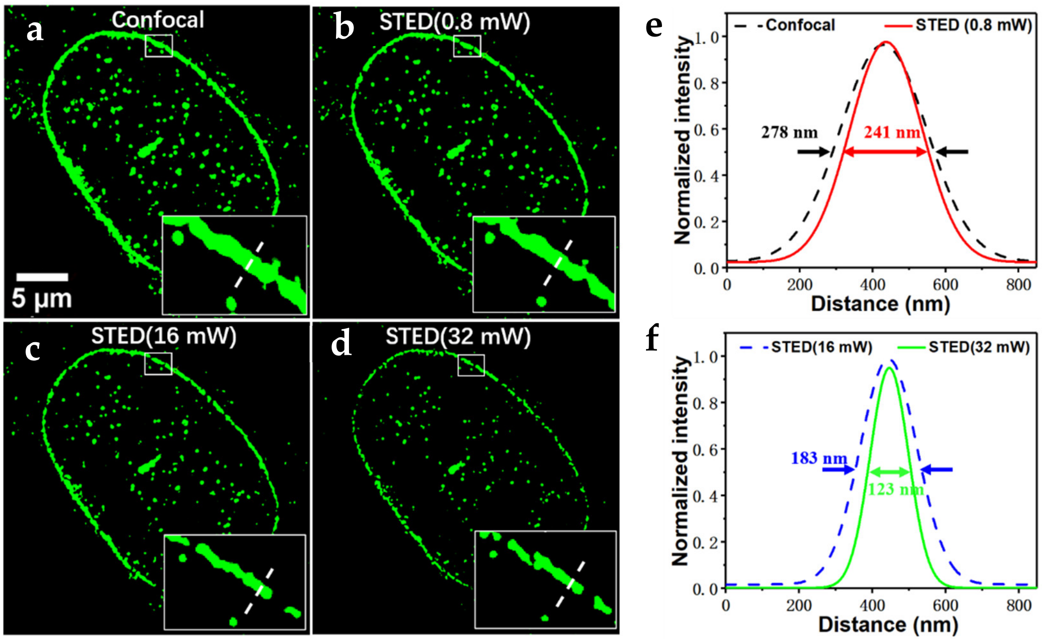

Figure 1. The comparison in resolution between confocal and STED images at different depletion powers of 488 nm channel. (a) Confocal image, (b)-(d) STED images obtained at depletion power of 0.8 mW, 16 mW and 32 mW. (e) Normalized intensity profiles along the white dashed lines in a and b for confocal (black curve) and STED (red curve) images; (f) Normalized intensity profiles

along the white dashed lines in c and d for 16 mW (blue curve) and 32 mW (green curve) images; Zoomed views of the boxed regions in white are shown in the insets. Scale bar: 5 μm.

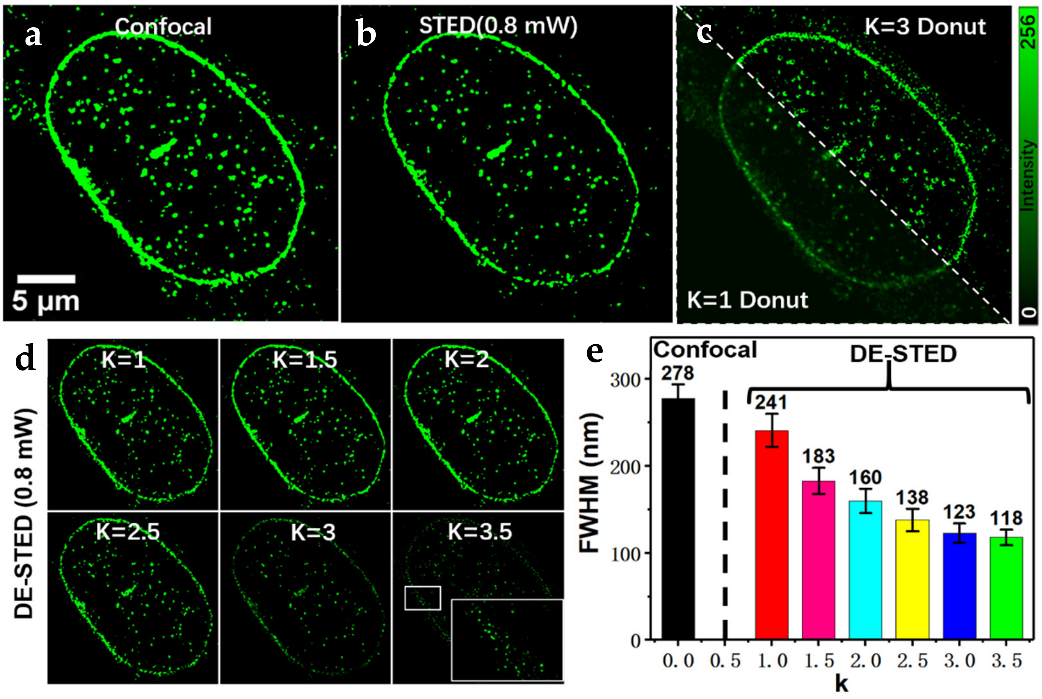

Figure 2. The images of nuclear envelope in a fixed U2OS cell in 488 nm channel. (a) Confocal image, (b) STED image at depletion power of 0.8 mW. (c) Donut image with k value of 1 and 3. (d) DE-STED images with the increase of k value. (e) Dependence of average FWHM of nuclear envelope on the k value. Scale bar: 5 μm.

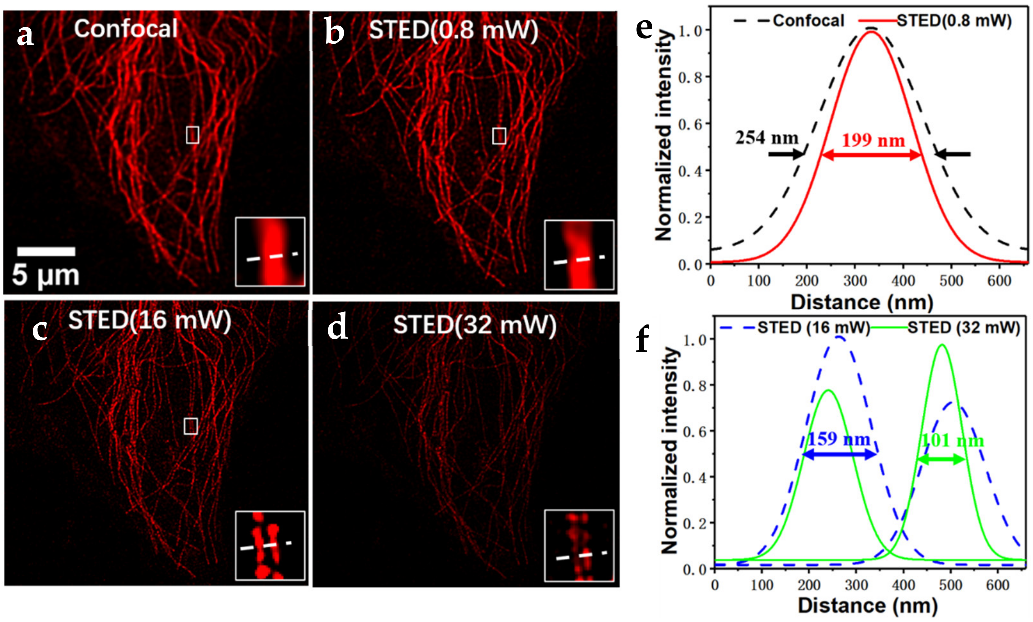

Figure 3. The comparison in resolution between confocal and STED images at different depletion powers in 635 nm channel. (a) Confocal image, (b)-(d) STED images obtained at depletion power of 0.8 mW, 16 mW and 32 mW. (e) Normalized intensity profiles along the white dashed lines in a and b for confocal (black curve) and STED (red curve) image; (f) Normalized intensity profiles along the white dashed lines in c and d for 16 mW (blue curve) and 32 mW (green curve) image; Zoomed views of the boxed regions in white are shown in the insets. Scale bar: 5 μm.

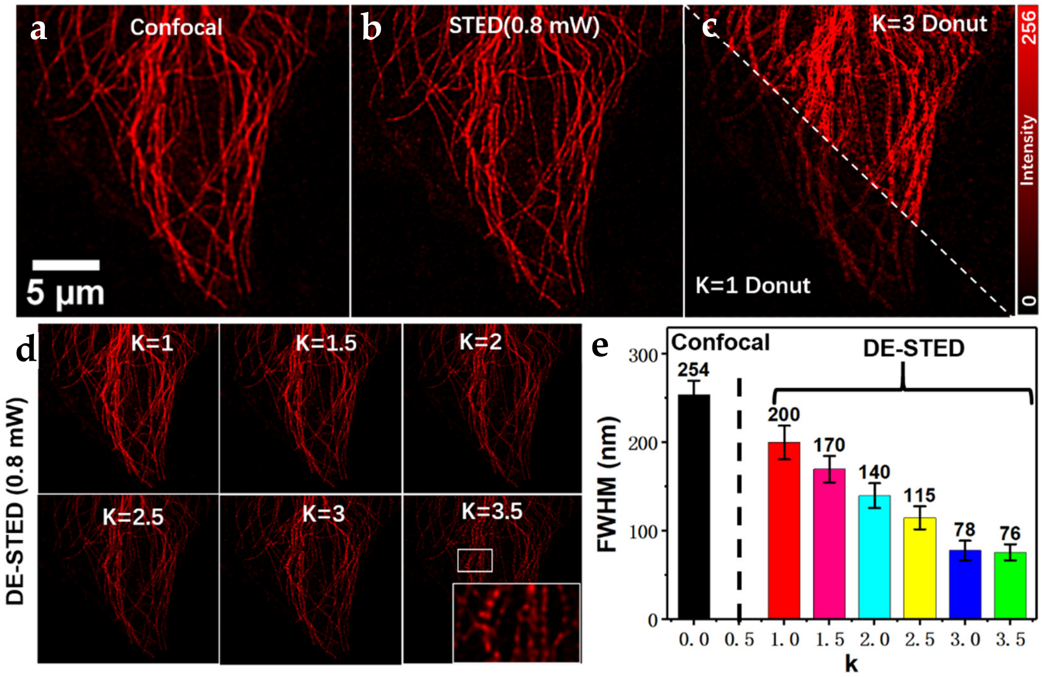

Figure 4. The imaging of tubulin in a fixed U2OS cell in 635 nm channel. (a) Confocal image, (b) STED image at depletion power of 0.8 mW. (c) Donut image with k value of 1 and 3. (d) DE-STED images with the increase of k value. (e) Dependence of average FWHM of tubulin on the k value. Scale bar: 5 μm.

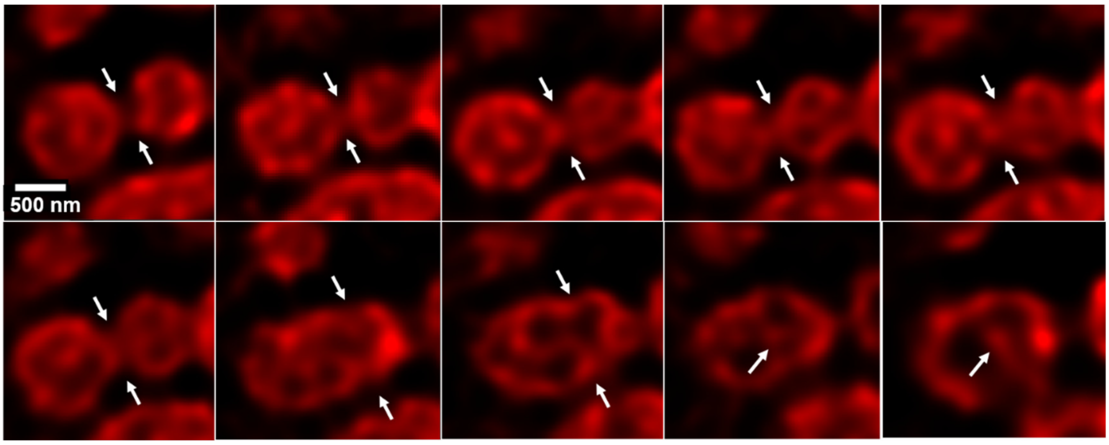

Figure S5. Long-term dynamic imaging of U2OS cell mitochondria in STED mode，the white arrow indicates a typical mitochondrial fusion process, one frame every 3 minutes. Scale bar: 500 nm.

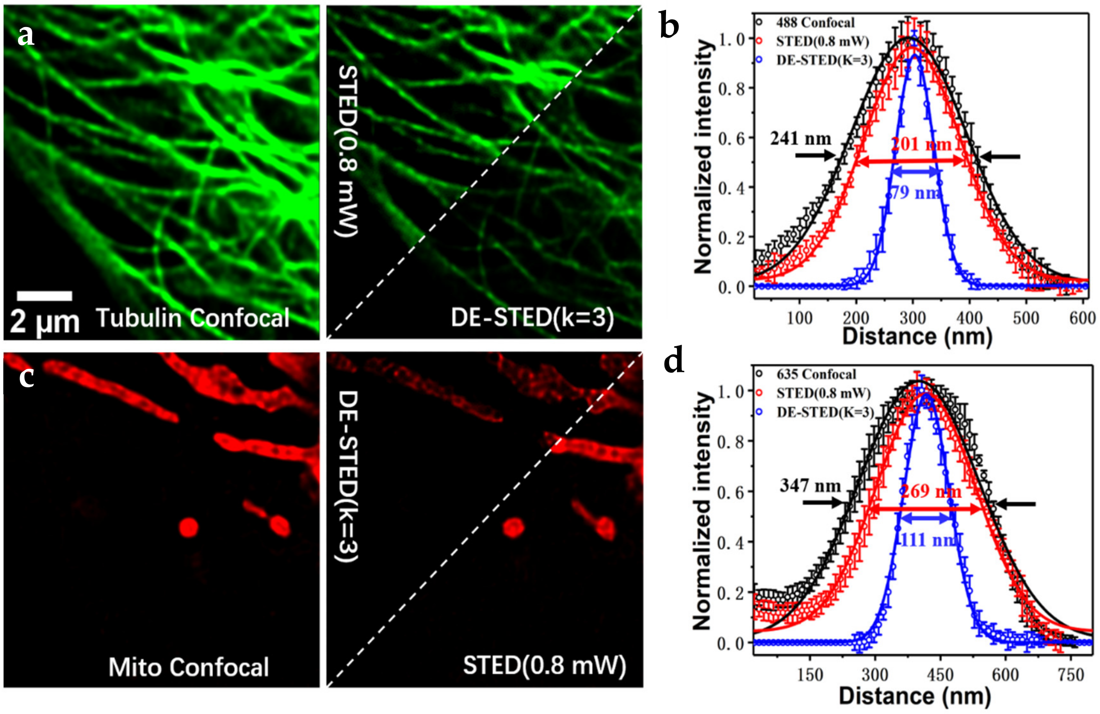

Figure 6. The comparison in resolution between confocal, STED, and DE-STED images of living U2OS cell. (a) Confocal, STED, and DE-STED(k=3) images of tubulin; (b)Mean cross section of tubulin in (a); (c) Confocal, STED, and DE-STED(k=3) images of mitochondrial; (d)Mean cross section of mitochondrial in (c). Scale bar: 2 μm.
